# Interventions Using Heart Age for Cardiovascular Disease Risk Communication: Systematic Review of Psychological, Behavioral, and Clinical Effects

**DOI:** 10.2196/31056

**Published:** 2021-11-05

**Authors:** Carissa Bonner, Carys Batcup, Samuel Cornell, Michael Anthony Fajardo, Anna L Hawkes, Lyndal Trevena, Jenny Doust

**Affiliations:** 1 School of Public Health Faculty of Medicine and Health University of Sydney Sydney Australia; 2 National Heart Foundation of Australia Brisbane Australia; 3 School of Public Health Faculty of Medicine University of Queensland Brisbane Australia

**Keywords:** heart age, cardiovascular disease, risk assessment, risk communication, prevention

## Abstract

**Background:**

Cardiovascular disease (CVD) risk communication is a challenge for clinical practice, where physicians find it difficult to explain the absolute risk of a CVD event to patients with varying health literacy. Converting the probability to *heart age* is increasingly used to promote lifestyle change, but a rapid review of biological age interventions found no clear evidence that they motivate behavior change.

**Objective:**

In this review, we aim to identify the content and effects of heart age interventions.

**Methods:**

We conducted a systematic review of studies presenting heart age interventions to adults for CVD risk communication in April 2020 (later updated in March 2021). The Johanna Briggs risk of bias assessment tool was applied to randomized studies. Behavior change techniques described in the intervention methods were coded.

**Results:**

From a total of 7926 results, 16 eligible studies were identified; these included 5 randomized web-based experiments, 5 randomized clinical trials, 2 mixed methods studies with quantitative outcomes, and 4 studies with qualitative analysis. Direct comparisons between heart age and absolute risk in the 5 web-based experiments, comprising 5514 consumers, found that heart age increased positive or negative emotional responses (4/5 studies), increased risk perception (4/5 studies; but not necessarily more accurate) and recall (4/4 studies), reduced credibility (2/3 studies), and generally had no effect on lifestyle intentions (4/5 studies). One study compared heart age and absolute risk to fitness age and found reduced lifestyle intentions for fitness age. Heart age combined with additional strategies (eg, in-person or phone counseling) in applied settings for 9582 patients improved risk control (eg, reduced cholesterol levels and absolute risk) compared with usual care in most trials (4/5 studies) up to 1 year. However, clinical outcomes were no different when directly compared with absolute risk (1/1 study). Mixed methods studies identified consultation time and content as important outcomes in actual consultations using heart age tools. There were differences between people receiving an older heart age result and those receiving a younger or equal to current heart age result. The heart age interventions included a wide range of behavior change techniques, and conclusions were sometimes biased in favor of heart age with insufficient supporting evidence. The risk of bias assessment indicated issues with all randomized clinical trials.

**Conclusions:**

The findings of this review provide little evidence that heart age motivates lifestyle behavior change more than absolute risk, but either format can improve clinical outcomes when combined with other behavior change strategies. The label for the *heart age* concept can affect outcomes and should be pretested with the intended audience. Future research should consider consultation time and differentiate between results of older and younger heart age.

**International Registered Report Identifier (IRRID):**

NPRR2-10.1101/2020.05.03.20089938

## Introduction

### Background

Cardiovascular disease (CVD) risk communication is a challenge for clinical practice, where physicians find it difficult to explain the absolute percentage risk of a CVD event to patients with varying health literacy [1,2]. Absolute risk calculators are recommended in clinical guidelines around the world as the best way to predict the risk of a CVD event over a relatively short period, by incorporating both modifiable (eg, smoking, blood pressure, and cholesterol levels) and nonmodifiable (eg, age and gender) risk factors [3-6]. These calculators are designed to determine whether preventive medication should commence, which is generally recommended for high risk and low risk categories. They are not designed to motivate or determine when to commence lifestyle changes, as this is recommended for all risk categories. For example, smoking cessation should always be recommended for a person who smokes, regardless of the calculated absolute risk. However, the implementation of absolute risk calculators has been poor, and communication barriers have been identified as one reason for this [2,7-10]. One proposed solution is to use more intuitive and motivating risk communication concepts rather than abstract probabilities [11,12]. *Heart age* has been suggested as an alternative to absolute percentage risk of a CVD event and can be calculated by comparing an individual’s absolute risk over 5 or 10 years with ideal risk factors (eg, blood pressure of 120/80 mm Hg) or the average for their age or gender category [11].

Heart age tools are increasingly used to promote behavior change around the world, including clinical contexts and web-based consumer resources. They have not generally been used to guide decisions to commence medication in the same way that absolute risk calculators have, although the Joint British Societies (JBS)-3 guidelines in the United Kingdom do suggest that older heart age may be considered as a reason to initiate medication [3]. Following clinical trials of the lung age and heart age concepts, the World Heart Federation collaborated with Unilever for an international promotion to at least 2.7 million consumers in 2009 [13]. Since then, heart age tools have also been promoted to support clinical guidelines in the United Kingdom, New Zealand, and Australia, reaching millions more through nonprofits and health services [14,15]. However, despite this mass appeal, there is no clear evidence that such tools actually motivate behavior change. A review of CVD risk communication in 2011 identified heart age as a potentially useful concept that requires further research [12]. A 2016 review of vascular age concepts in clinical applications found limited trials testing the effects of communicating this concept [16], but a recent rapid review in 2020 highlighted the increasing number of studies on age-based risk formats, suggesting there may now be more evidence available [17].

Heart age tools vary widely in terms of their underlying risk models and results, with the possibility of receiving an older heart age on one calculator but a younger result on another [18]. This is because the tools have different underlying algorithms to assess CVD event risk (eg, Framingham vs QRISK), and different thresholds for comparing an individual’s result with ideal or average risk factors (eg, systolic blood pressure may be compared with 120 for ideal vs 125 for average). Another factor is that many people do not know their blood pressure or cholesterol levels, and different techniques are used to estimate this (eg, BMI or population average) [13].

In addition to the underlying algorithms, the way heart age results are explained can come in many forms. The Australian heart age calculator is relatively simple with a single heart age result and prompts an individual to see a physician for a more accurate risk assessment [14]. UK-based heart age calculators include numerous risk communication formats, including the percentage chance of a heart attack or stroke over 10 years or a lifetime, estimated life expectancy, and graphical displays [15]. Heart age tools are often linked to further lifestyle change messages [14], but research on heart age interventions has not differentiated well between these components, despite the fact that they represent different behavior change techniques—the *active ingredients* of behavioral interventions [19]. Heart age interventions may range from a simple one-off message frame (eg, communicating risk as *older heart age* without any further information) to complex programs involving health professional counseling and goal-setting or monitoring of heart age over time.

### Objectives

Previous reviews relating to the heart age concept have been descriptive about the models [16,20] or concepts but have not made a distinction between the comparison groups involved in trials (eg, heart age vs standard care or absolute risk [12,17]), have not clearly identified the behavior change techniques included within heart age interventions [12,17,20], or have not included qualitative studies that may provide additional insights into why these tools are so widely used in spite of limited evidence for their effectiveness [16,17,20]. The aim of this systematic review is to identify the content and effects of heart age interventions presented to patients or consumers for the purpose of CVD risk communication in detail, in order to shed light on mixed evidence of their effectiveness.

## Methods

Our methods protocol was prepublished on the preprint server medRxiv [21] (not peer-reviewed).

### Eligibility Criteria

Studies were considered eligible if they met the following criteria:

1. Published from the inception of the database to April 2020 (this was later updated to March 2021) in peer-reviewed journals.
2. Population: used an adult population (over 18 years of age) or, if not explicit regarding age, are clear that participants were not children.
3. Intervention: present the concept of *heart age* to patients or consumers for the purpose of CVD risk communication, in any setting, including general practices, hospitals, health clinics or community centers, workplaces, or on the web. This included both simple message frame experiments and complex programs in applied settings.
4. Comparators: we placed no restrictions on whether a comparison or control group was used.
5. Outcomes: report qualitative themes or quantitative outcomes related to psychological or behavioral responses to heart age, including clinical outcomes.

Studies that were not peer-reviewed journal articles, such as conference proceedings, dissertations, or government reports, were excluded. Protocol papers, opinion papers, reviews, web-based user descriptions, and heart age algorithm development or validation were excluded. Some studies applied a heart age algorithm to population data or individual patients as an outcome, but the results were not conveyed to individuals, so these were excluded. Studies that presented heart age to adults but did not measure outcomes or collect qualitative data were also excluded.

### Information Sources

The following databases were searched: the Cochrane Central Register of Controlled Trials (via OvidSP), MEDLINE (via OvidSP), Embase (via OvidSP), and PsycINFO (via OvidSP) up to March 2021. The search terms are based on an earlier vascular age review in 2016, with additional free text terms based on known relevant papers. The full list of terms is based on a previous review and includes (*vascular*, *vessel*, *arterial*, *heart*, *cardiovascular*, *coronary*, *risk* AND *age*, *ages*, *ageing*, or *aging*), OR *heart forecast*, and limited to human studies. We then searched the citations and references of the final included studies and 2 previous reviews of vascular age models and more general age-related risk concepts. We also included any papers mentioned on publicly accessible heart age websites.

### Data Management

We downloaded the references identified in the searches (electronic databases and additional searches) into Microsoft Excel. Duplicates were then removed, and 2 reviewers (SC and C Batcup) screened the titles and abstracts of each study to determine whether they should be included. Discrepancies were resolved by discussion with C Bonner.

### Selection Process

The screening process was undertaken by 2 review authors (C Batcup and SC). Each reviewer independently assessed a study’s suitability to be included in the review by marking against each study on an Excel spreadsheet, which contained the title and abstract. Studies that did not meet the inclusion criteria were excluded. We obtained the full text of the remaining papers and then assessed the remaining papers against the full inclusion terms for the review to determine their eligibility for inclusion. Non–English language papers were translated into English using Google Translate and verified for inclusion or exclusion by speakers of the relevant language. The review authors resolved disagreements through a consensus-based decision-making process or, when necessary, discussion with a third review author (C Bonner). Two Japanese language studies were considered for inclusion but deemed ineligible by an author who could read Japanese (JD).

### Data Extraction

Two review authors (C Batcup and SC) completed web-based training to apply the behavior change technique taxonomy to published methods and used a predefined data extraction form to collect data from the included studies. Reviewers piloted the data extraction form with a sample of included papers; however, no amendments were made. An Excel database was used to extract quantitative and qualitative data from the included studies.

*Quantitative data extracted* included year, country, study design, study population (age, education, socioeconomic status, health literacy, race or culture or ethnicity), number of participants, intervention format (web or paper based), comparison groups (standard care or absolute risk alone), clinical measures (blood pressure, cholesterol levels, weight, BMI, waist circumference, and prescribed medications), behavioral measures (medication adherence, lifestyle intentions or self-report), psychological measures (probabilistic or evaluative risk perceptions, positive or negative emotional responses, credibility, and recall), and a summary of significant effects of heart age communication.

*Qualitative data extracted* included behavioral themes (eg, lifestyle change), psychological themes (eg, emotional reaction), stated benefits of heart age (eg, motivates people to take action), and stated problems with heart age (eg, reduced credibility).

*Intervention content data* included additional risk communication formats (eg, absolute risk, risk level, graphs), underlying model (eg, 5-year or 10-year CVD risk model), and behavior change techniques (eg, email prompts or action plans) coded based on the taxonomy of 93 techniques by Michie et al [19]. This was done based on the methods published in the results paper and any referenced protocol papers.

*Risk of bias assessment* included randomized studies that were critically appraised independently by 2 review authors (MF, C Bonner, or C Batcup if C Bonner was an author) using the relevant Johanna Briggs Institute tools for the study design [22]. Disagreement was resolved by discussion, with decisions applied consistently where there was a common methodology, for example, if the participants were randomized on a computer, *was allocation to groups concealed* was marked as not applicable (N/A). Similarly, as all participants in all the papers could see the risk communication format they were allocated to, all were marked as *No* for being *blind to treatment assignment*.

## Results

### Overview

**Figure 1:**
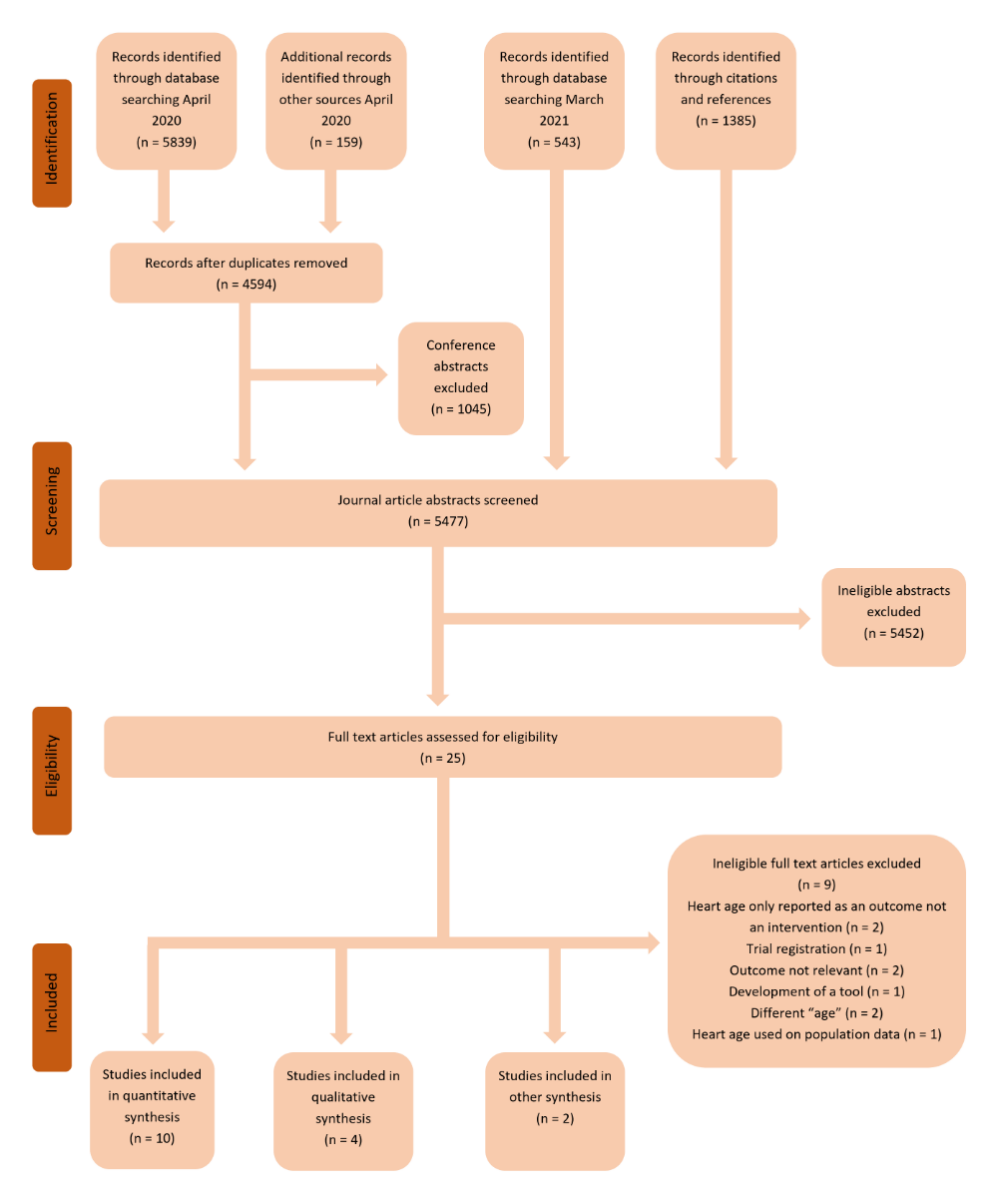
PRISMA (Preferred Reporting Items for Systematic Reviews and Meta-Analyses) diagram.

Figure 1 shows the search process and results. In April 2020, 5839 database results and 159 references from previous reviews were assessed for eligibility, and in March 2021, an additional 543 database results and 1385 citations and references were reviewed. From 7626 total results, 16 eligible studies were identified with heterogeneous study designs and outcomes, and the results are reported below by study type: 5 randomized web-based experiments comparing heart age with percentage risk, 5 randomized clinical trials with mixed interventions, 2 mixed methods studies with quantitative outcomes, and 4 qualitative studies. Study details and outcomes are summarized by study category in Tables 1-3. Multimedia Appendix 1 [23-32] provides details about the measures used in each study, and Multimedia Appendix 2 [14,23-37] provides details of behavior change techniques included in the control versus intervention groups.

**Table 1:** Randomized web-based experiments directly comparing heart age with absolute risk.

Study details	Intervention format	Comparison groups	CVD^a^ risk algorithm	Participants	Principal findings
Soureti et al (2010) [23]; 2-arm RCT^b^ in the United Kingdom	Web-based questionnaire, postintervention outcomes	1. 10-year percentage risk 2. Heart age	Framingham	413; 209 in percentage risk, 204 in heart age; aged 30-60 years	Intentions to change behavior: no significant differences in intention to stop smoking, improve diet, or increase physical activity between heart age and percentage risk groups. Higher worry and identifying the information as a wake-up call were significantly correlated with overall intention to change behavior. Risk perceptions: no difference in average risk perception between heart age and percentage risk groups. Higher worry and identifying the information as a wake-up call were significantly correlated with risk perceptions. Emotional response: no difference in terms of levels of worry or perceiving the information as a wake-up call between heart age and percentage risk groups. For younger participants with higher levels of risk, the heart age group was more likely to have a worried response and perceive the message to be a wake-up call than the percentage risk group. The 2 items were also highly correlated. Credibility: no difference in credibility between heart age and percentage risk groups.
Witteman et al (2014) [24]; 2-arm RCT in the United States	Web-based questionnaire, postintervention outcomes	1. 10-year percentage risk 2. 10-year percentage risk and heart age	Framingham	3630, numbers in each group not given; aged 35-74 years; mean 53 (SD 10) years	Intentions to change behavior: no difference between heart age and no heart age on quitting smoking, exercising, eating a DASH^c^ diet, losing weight, and seeing a physician in the next 30 days.
Bonner et al (2015) [25]; 2 × 3 factorial design RCT in Australia	Web-based questionnaire. Participants shown either 5-year absolute risk or heart age, and within that different text and visual formats. Postintervention outcomes and followed up on the web 2 weeks later	1. 5-year percentage risk: (a) text only; (b) text + bar graph; (c) text + line graph 2. Heart age: (a) text only; (b) text + bar graph; (c) text + line graph	Framingham	570; 281 in percentage risk and 289 in heart age; aged 45-64 years; mean 54 (SD 6) years	Intentions to change behavior: for intention to change lifestyle (diet, physical activity, smoking, and the average of these), there were no significant differences between the heart age and percentage risk groups. Self-reported behavior change: at 2-week follow-up, no differences were found between heart age and percentage risk groups (adequate diet, adequate physical activity, smokers, or making a GPd appointment for CVD risk assessment). Risk perceptions: heart age was more likely to be perceived as indicating moderate or high risk compared with percentage risk, even though the sample was predominantly low risk (*P*<.001). Emotional response: the heart age group had a less positive emotional response to the risk result compared with the percentage risk group (*P*<.001). No difference in negative emotional response. Credibility: lower perceived credibility for the heart age group vs the percentage risk group (*P*<.001). Recall: there was no difference in recall immediately postintervention. However, those in the heart age group are significantly more likely to correctly recall their exact result after 2 weeks (32%) vs percentage risk group (16%; *P*<.001).
Damman et al (2018) [26]; 2×2 factorial design RCT in the Netherlands	Web-based questionnaire (hypothetical results). Postintervention outcomes	1. Infographics of 10-year risk information (a) alone or (b) with a risk percentage and icon array 2. Text of risk information (a) alone or (b) with a risk percentage and icon array 3. Heart age with infographics	Framingham	727; 151 in infographics alone, 145 in infographics plus risk percentage, 133 in risk text alone, 168 in risk text plus risk percentage, 130 in heart age; aged 45-65 years	Intentions to change behavior: mixed results: intention to visit GP (*P*=.02) and to become more physically active (*P*=.01) were significantly different between percentage risk and heart age (more likely to if seeing heart age) but no difference for eating more healthily and using medication. Risk perceptions: heart age perceived risk as higher: more likely to experience a CVD event (*P*=.003), saw their risk as higher chance (*P*=.004), and overall comprehended their risk as higher (eg, serious consequences, means something is going on with my health; *P*=.02). Credibility: in terms of thinking the information is clear, relevant, useable, realistic, etc, no difference between percentage risk and heart age, apart from the fact that the information is helpful (*P*=.03). Emotional response: worry was significantly higher in the heart age group (*P*=.02), positive affect was no different, and negative affect was significantly higher in the heart age group (*P*=.002). Recall: those with heart age were correct in recalling their heart age 60.8% of the time vs 55.2% of the time for percentage risk (not a significant difference). However, the heart age group was significantly (*P*=.008) more likely to recall the verbal label (increased risk—66.2% vs 50.3%). There were no significant differences in recall of the causes, timeline, or consequences of their risk result.
Van Der Pol-Harney et al (2021) [27]; 2 × 3 factorial design RCT in Australia	Web-based questionnaire. Participants randomized to one of 3 risk communication formats and received low or high risk based on self-report lifestyle risk factors. Postintervention outcomes	1. Lifetime percentage risk 2. Heart age 3. Fitness age	Provided with either low (5%, 16 years) or high (69%, 35 years) lifetime risk. High risk=smoke or eat 1 or no servings of fruit per day	174; 53 in percentage risk, 50 in heart age, 71 in fitness age; mean age 19 (SD 2.3) years	Intentions to change behavior: fitness age group had lower intentions to change diet and exercise than the heart age group (*P*=.048), percentage risk group (*P*=.02), and these 2 groups combined (*P*=.02). Risk perceptions: receiving a high-risk result was associated with higher perceived numerical, verbal, and comparative risk (across all formats). Perceived numerical and comparative risk did not vary greatly with actual risk for those given a fitness age; however, those given either a heart age or percentage risk format expressed higher perceived risk after being categorized as high risk. Emotional response: receiving a high-risk result was associated with greater postintervention worry (for all formats), more so for smokers. Credibility: receiving a high-risk result was associated with lower credibility, across all risk formats. This difference was greatest in the heart age group. Results were more likely to be seen as credible for participants who received results better than expected.

^a^CVD: cardiovascular disease.

^b^RCT: randomized clinical trial.

^c^DASH: Dietary Approaches to Stop Hypertension.

^d^GP: general practitioner.

**Table 2:** Randomized clinical trials in applied settings comparing mixed interventions.

Study details	Intervention format	Comparison groups	CVD^a^ risk model	Participants	Principal findings
Lowensteyn et al (1998) [28]; RCT^b^ in Canada	Physicians enrolled their own patients who they thought would benefit from a risk profile. Followed up 3 months later	1. Control: usual care 2. Intervention: paper-based risk profile, including their 8-year risk of developing coronary disease, and how this risk would reduce if they changed one or more risk factors. Cardiovascular age also shown.	Framingham	958; 176 in control, 782 in risk profile; aged 30-74 years; mean age 51 (SD 11) years	Blood pressure: no difference between change in blood pressure in profile group vs control group (–2 systolic in profile group vs –1.2; –0.9 for diastolic in profile group vs 0.1). Cholesterol: at the 3-month follow-up, patients who were shown their risk profile had significantly greater reductions (*P*<.05) in total cholesterol, LDLc, and total or HDLd cholesterol ratio (after adjusting for group differences at baseline and clustering for same physician). Absolute risk: Significantly greater improvement in cardiovascular age (*P*<.01) and 8-year coronary risk (*P*<.01) compared with the control group (because of cholesterol change). Weight: no difference in BMI between groups.
Grover et al (2007) [29]; RCT in Canada	Physicians enrolled their patients. Baseline visit, and followed up at 3, 6, 9, and 12 months	1. Control: usual care 2. Intervention: paper-based risk profile including cardiovascular age, ongoing feedback on risk after lifestyle changes or medication	Framingham (or Cardiovascular Life Expectancy Model for patients with CVD)	3053 received initial intervention: 1510 in risk profile group and 1543 in control; mean age 64 (SD 8) years	Blood pressure: after 12 months, both systolic (*P*=.005) and diastolic (*P*=.01) blood pressure decreased significantly more in the intervention group vs usual care. Cholesterol: patients who were shown their risk profile reduced their LDL cholesterol by 51.2 mg/dL whereas in usual care it reduced by 48.0 mg/dL (*P*=.02). Similarly, total cholesterol (*P*=.02) and cholesterol ratio (*P*=.002) was significantly more reduced in the intervention group at 1 year. HDL cholesterol was no more improved in the risk profile group than in the control group. Absolute risk: significantly greater improvement in 10-year risk of CVD in the risk profile group 12 months later (*P*<.001).
Lopez-Gonzalez et al (2013) [30]; RCT in Spain	Participants interviewed by researchers and clinical assistants; measurements taken. Follow-up measurements taken 12 months later	1. Usual care 2. Percentage risk 3. Heart age	Framingham	2844: 975 in usual care, 955 in percentage risk, and 914 in heart age; mean age 46 (SD 7) years	Self-reported behavior change: physical activity sessions per week decreased in control (0.35) but increased to a similar extent in both risk (0.68) and heart age groups (0.88; all *P*<.001). Number of people currently smoking increased in control by 0.9%, decreased in risk by 0.4%, and decreased in heart age by 1.8% (*P*<.001). Blood pressure: systolic blood pressure reduced by 2.31 mm Hg in risk vs 4.37 mm Hg in heart age, diastolic reduced by 1.77 mm Hg in risk and 2.88 mm Hg in heart age. Control increased in both (1.02 systolic and 1.21 diastolic; *P*<.001). Cholesterol: total reduced by 3.36 mg/dL in percentage risk, 6.54 mg/dL in heart age. HDL increased by 0.47 mg/dL in risk and 1.27 mg/dL in heart age. Triglycerides reduced by 2.65 mg/dL in risk and 5.14 mg/dL in heart age. Control increased in both total (5.36) and triglycerides (4.38) and decreased in HDL (0.92; all *P*<.001). Weight: weight decreased by 0.22 kg in risk, 0.77 kg in heart age. Control increased by 0.72 kg (*P*<.001). BMI reduced by 0.11 in risk, 0.27 in heart age. Control increased by 0.25. Overall difference between 3 groups (*P*<.001). Waist circumference reduced by 0.05 cm in risk, 0.15 cm in heart age. Control increased by 0.13 cm (*P*<.001).
Näslund et al (2019) [31]; RCT in Sweden	Participants meeting with their primary care physician, measurements taken. Follow-up measurements taken 12 months later	1. Control: completing a primary care health survey including CVD risk factor screening, pharmacological CVD prevention if required, and advice on healthy lifestyle, and an ultrasound 2. Intervention: the above plus a pictorial representation of carotid ultrasound (including vascular age) plus a nurse phone call to confirm understanding 2-4 weeks later and information repeated after 6 months	Framingham	3532; 1783 in control, 1749 in intervention; aged 40-60 years	Self-reported behavior change: significant increase in use of lipid-lowering medication in the intervention group compared with control group (*P*<.05). Blood pressure: systolic increased by 1.6 mm Hg in control and was stable (–0.2 mm Hg) in the intervention group—not significant. Cholesterol: total and LDL decreased in both groups, but the reduction was greater in the intervention group than in the control group at the 1-year follow-up (*P*<.05). Weight: slight increase in control group and slight decrease in intervention group—not significant. Absolute risk: at the 1-year follow-up, those in the intervention group had a decreased Framingham risk score, whereas in the control group this was increased (*P*<.05). Systematic coronary risk evaluation measure increased to a lesser extent in the intervention group (*P*<.05).
Svendsen et al (2020) [32]; cluster RCT in Norway	Participants discussed risk with pharmacy staff. Follow-up after 4 weeks	1. Control: conventional risk communication, each risk factor categorized in 4 groups from good (green) to poor (red), and diet and lifestyle advice given verbally and in written form 2. Intervention: the above plus heart age	JBS^e^-3	257; 120 in control, 137 in intervention; mean age 60 (SD 13) years	Self-reported behavior change: physical activity levels did not change after 4 weeks in either of the groups. Blood pressure: no differences in blood pressure levels. Cholesterol: no differences in cholesterol levels between the groups. Consultation communication: the heart age tool was considered a convenient and motivating communication tool by pharmacy staff.

^a^CVD: cardiovascular disease.

^b^RCT: randomized clinical trial.

^c^LDL: low-density lipoprotein.

^d^HDL: high-density lipoprotein.

^e^JBS: Joint British Societies.

**Table 3:** Mixed methods studies with no randomization.

Study details	Intervention format	Comparison groups	CVD^a^ risk model	Participants	Principal findings
Goldman et al (2006) [34]; focus groups in the United States (qualitative)	Responded to 3 visual representations of risk (all of a hypothetical man aged 42 years)	1. Icon chart risk vs ideal 2. Bar chart risk vs ideal 3. Bar chart heart age vs ideal vs age	Hypothetical person but based on Framingham	50 adults in 7 focus groups; aged 27-84 years	Emotional response: bar graph lacked impact: it was “too statistical,” “scientific,” “too dry.” But heart age was “catchy,” memorable, and engaging. Some participants said patients may be alarmed by heart age. Debate as to whether it is motivating or just frightening. Still thought heart age was better though as more engaging and memorable Credibility: some skepticism about the validity of age calculation
Bonner et al (2014) [36]; think-aloud process and interviews in Australia (qualitative)	Participants viewed 2 different heart age calculators	1. Heart age 2. 10-year percentage risk and heart age	Framingham	26 patients recruited from general practice; aged 39-67 years	Intentions to change behavior: heart age calculators led participants to consider lifestyle changes Emotional response: heart age elicited emotional responses; for example, younger heart age seen as positive and older heart age was confronting Credibility: process of using the calculators results in different credibility perception Understanding: not understanding percentage risk information, but heart age much easier to understand and more meaningful Other: modifying risk factors had mixed response; for example, some not interested or did not understand and some spent time changing things
Shefer et al (2016) [35]; interviews and focus groups in the United Kingdom (qualitative)	Patients randomized to different web-based questionnaires. Then either interviewed or took part in a focus group	1. Control 2. Lifestyle advice only 3. Lifestyle advice plus 10-year percentage risk (phenotype) 4. Lifestyle advice plus 10-year percentage risk (phenotype + genetic) *includes heart age and ideal risk*	Framingham	41 adults in interviews (22 in group 4, 15 in group 3, and 4 in group 2) and 13 adults in 2 focus groups (one with 6 patients and one with 7; 8 in group 4, 5 in group 3); aged 40-80 years	Intentions to change behavior: for some, heart age was a “wake-up call” to make changes. Self-reported behavior change: more than two-thirds, including those with low or medium risk, maintained lifestyle changes (gap between seeing the intervention and interview was between 1 and 134 days)—although modest. Intervention added as a “reminder,” “trigger”—already aware they needed to do something beforehand. Risk perceptions: despite two-thirds having an older heart age, only a minority were concerned about their risk. Could be because of not recollecting their risk score, or not remembering context of whether the percentage risk was low or high even if they did remember the number. Or that they overestimated their risk before the intervention (eg, female mean risk of 3.5% but mean predicted risk of 29.5%). Or that many patients thought a high risk was above 50%, so lower than that did not seem that high; one-fourth concerned about risk, all of them concerned primarily with heart age, despite having a risk above 20%. Emotional response: heart age stood out as a powerful message about patients’ lifestyle: “it was the heart age that really shook me.” Link to age; for example, “risk is that of somebody who’s retired.”
Riley et al (2020) [37]. Recorded GP^b^ consultations in the United Kingdom (qualitative)	GP consultations using either JBS^c^-3 or QRISK calculators were recorded and analyzed qualitatively	1. JBS-3 calculator 2. QRISK calculator	JBS-3 or QRISK	128 consultations analyzed; 64 in QRISK group and 64 in JBS-3; aged 40-74 years	Intentions to change behavior: coping appraisal more common in JBS than QRISK. Not much discussion around costs for changing behavior. Some maladaptive coping; for example, dismissive of suggestions. Sometimes maladaptive responses to the percentage risk score could be prompted into a more positive response through communication of heart age. Adaptive coping shown by a number of patients showed intentions to change behavior as a result of seeing their risk Risk perceptions: threat appraisal observed in all consultations (although less frequently in JBS-3 consultations vs QRISK). Patients acknowledged their risk level but understanding of percentage risk was unclear. Heart age aided understanding and intention to change risk Credibility: surprised at their risk leading to questioning how the risk was calculated Consultation communication: misunderstanding of risk, which was not helped by the GP, although more evidence of active practitioner-patient engagement in the JBS-3 group following risk score manipulation. GPs seemed less confident in discussing percentage risk than heart age. GPs consistently did not ask questions to check understanding. Understanding: understanding of 10-year percentage risk was unclear. Heart age aided patient understanding of CVD risk.
Gidlow et al (2020) [33]. Recorded GP consultations in the United Kingdom (quantitative)	GP consultations using either JBS-3 or QRISK calculators were recorded	1. JBS-3 calculator 2. QRISK calculator	JBS-3 or QRISK	173 general practice consultations; 73 QRISK and 100 JBS-3; aged 40-74 years	Consultation time: 10% of time discussing CVD risk in JBS-3 vs 7% in QRISK. 35% (JBS-3) vs 41% (QRISK) of time spent discussing CVD risk factors. Risk management interventions discussed in 19% of JBS-3 vs 21% of QRISK. Lifestyle interventions discussed in 16% of JBS-3 and 18% of QRISK. Medication in 58% of JBS-3 and 70% of QRISK Consultation communication: 94% vs 95% of consultations referenced the percentage risk score. Proportion of patients asking questions on risk was higher in JBS-3 than QRISK (32% vs 12%). All physicians discussed heart age in JBS-3 vs 52% in QRISK. Risk manipulation shown in 92% of JBS-3 and 22% of QRISK. Physicians spoke for 47% of time in JBS-3 and 55% in QRISK. Verbal dominance ratio of 2.35 in JBS-3 and 3.21 in QRISK
Bonner et al (2020) [14]; survey in Australia (quantitative outcomes and content analysis of open responses)	Web-based heart age calculator open to the public. Some participants elected to receive their results via email. A subgroup completed a survey about their results, 10 weeks after seeing them	1. Heart age	Framingham	361,044 heart age calculator users; 30,279 provided email to receive heart age report; 1303 survey respondents; Mean age of users 49; of those who requested a report 56; survey respondents 60	Intentions to change behavior: Content analysis—either no motivation to change or it is a wake-up call to change lifestyle to reduce the heart age. Self-reported behavior change: 63% improved diet and 62% physical activity, 32% reduced stress, 31% reduced alcohol, 48% of smokers reduced. 48% saw GP and 28% had heart health check. Diet and seeing physician were more likely for older heart age than younger or equal heart age. Risk perceptions: Content analysis—whether heart age was higher or lower affects perception of risk. Emotional response: 39% very motivated, 25% very optimistic, 13% very anxious, 12% very worried. Older heart age associated with more anxiety or worry and less optimism, but similar motivation versus younger or equal heart age. Reflected in content analysis. Credibility: Content analysis—expectations affected credibility; for example, “I’m a bit unsure why as I exercise regularly,” “my cardiologist...said my heart is very good,” “questions were quite limited and did not take account lifestyle.” Recall: Most were able to recall their heart age category 10 weeks later (69%; although unclear if they accessed report again), especially for those with younger (67%) and older (70%) heart ages. It was lower for equal heart age results (57%). Cholesterol: 57% checked their cholesterol in the 10 weeks after seeing their heart age. More likely for those with older heart age. Weight: 49% reported weight loss 10 weeks after getting heart age. This was more significant for those with a higher heart age vs younger or equal heart age.

^a^CVD: cardiovascular disease.

^b^GP: general practitioner.

^c^JBS: Joint British Societies.

### Randomized Web-Based Experiments

Direct comparisons between heart age and absolute risk in 5 web-based experiments [23-27], with no in-person computer lab experiments identified. The studies included 5514 consumers and found that heart age leads to more negative emotional responses (3/4, 75% of relevant studies show higher negative emotions or lower positive emotions); higher perceptions of CVD risk being higher probability, more serious or in a higher risk category (2/4, 50% of relevant studies); higher exact or verbal recall (2/2, 100% of relevant studies); lower perceived credibility (2/3, 67% of relevant studies); and generally had no effect on lifestyle intentions (1/5, 20% of relevant studies) or self-reported behavior (no study). One study compared heart age and absolute risk to fitness age among young adults and found that fitness age led to lower lifestyle change intentions compared with the other formats [27]. All trials used the US Framingham model for risk except Van der Pol-Harney et al [27], which used lifetime CVD risk estimates and hypothetical ages to indicate low risk (younger age) and high risk (older age). All trials measured self-reported outcomes immediately postintervention, and 1 study conducted a follow-up survey after 2 weeks [25]. Soureti et al [23] compared the calculated 10-year risk to heart age among 413 people and found no significant differences for smoking, diet or exercise intentions, risk perception, emotional response, or credibility. They found that younger people with higher risk were more likely to be worried and perceive the result as a *wake-up call* when receiving heart age. Witteman et al [24] compared personalized 10-year risk to 10-year risk + heart age among 3630 people and found no significant differences in smoking, exercise, diet, weight, or physician visit intentions. Bonner et al [25] compared personalized 5-year risk to heart age with varying graphical formats in a 2 × 3 design among 570 people and found no significant difference in diet, exercise, smoking, or physician visit intentions or behavior after 2 weeks, or information seeking. They found that heart age led to lower positive emotions and credibility, and higher risk perception (such that low-risk people thought they were high risk) and recall after 2 weeks. Damman et al [26] compared hypothetical 10 year risk in various formats to heart age in a 2×2 design among 727 people and found mixed effects of heart age on intentions (higher intentions to visit a physician and exercise; no effect for diet or medication), higher risk perception, no difference in information perceptions relating to credibility (but heart age was perceived as more helpful), and higher recall for the verbal *increased risk* evaluative label. Van der Pol-Harney et al [27] compared the hypothetical lifetime risk to heart age or fitness age among 174 younger adults with different low- versus high-risk values for those with and without lifestyle risk factors in a 2 × 3 design. They found that heart age and percentage risk were generally equivalent, but there was a detrimental effect of fitness age, including lower exercise and diet intentions, lower credibility when given a high-risk result, and lower risk perception for high-risk results. Receiving a high-risk result was associated with higher risk perception, higher worry especially for smokers, and lower credibility, especially if the results were worse than expected (Table 1).

### Randomized Clinical Trials

When heart age was combined with additional strategies (eg, in person or phone counseling) in 5 applied trials [28-32] for 9582 patients, it improved risk control (eg, reduced cholesterol and absolute risk) compared with usual care in most trials (4/5, 80% of relevant studies) up to 1 year. However, the direction of outcomes (lifestyle, blood pressure, cholesterol, and weight) was the same for absolute risk and heart age groups in one trial that compared each group to usual care (1/1, 100% of relevant studies). Follow-up periods ranged from 4 weeks to 12 months, and all trials used the US Framingham model for risk except Svendsen et al [32], which used the UK JBS-3 model. Lowensteyn et al [28] compared a paper-based risk profile intervention (8-year risk, cardiovascular age, effect of reducing risk factors) to usual care among 958 patients over 3 months and found no difference in blood pressure but lower cholesterol, leading to lower absolute risk. Grover et al [29] compared a paper-based risk profile including cardiovascular age plus 3-monthly feedback to usual care among 2687 patients over 1 year and found lower blood pressure and cholesterol, leading to lower absolute risk. Lopez-Gonzalez et al [30] compared a web-based interactive heart age tool to verbal communication of percentage risk or usual care among 2844 employees over 1 year and found higher physical activity and lower smoking, blood pressure, cholesterol, weight, and waist circumference in intervention groups versus control, with greater benefits in the heart age group; however, analyses of the difference between heart age and risk groups were not reported. Näslund et al [31] compared 2 complex interventions, including one with heart age (heart age intervention: risk assessment and advice plus a carotid ultrasound image including vascular age and a nurse phone call, with information repeated after 6 months; control: risk assessment and advice only) among 3532 patients. They found higher use of cholesterol medication, lower cholesterol levels, and lower absolute risk in the heart age intervention group than in the control group; however, there was no difference in blood pressure or weight. Svendsen et al [32] compared conventional risk communication (risk categories, colors, evaluative labels, verbal and written advice) to the same information plus heart age among 257 patients visiting a pharmacy, and they found that although using heart age was popular among pharmacy staff, adding heart age was no more effective than conventional risk communication alone in changing physical activity or reducing cholesterol and blood pressure levels (Table 2).

### Mixed Methods Studies With Quantitative Outcomes

Two studies used a mixed methods design to investigate heart age: a survey of 1303 users of the Australian heart age calculator included quantitative outcomes and thematic content analysis of open responses [14]; and a UK study of video consultations using QRISK2 and JBS-3 risk calculators coded the content for quantitative outcomes in 12 general practices [33]. Because of their study design, it is not possible to attribute outcomes to heart age from these studies, but they do suggest additional possible outcomes of heart age tools: different effects for older versus younger or equal heart age results, and risk communication content and time within consultations. An Australian survey found that a subsample of heart age users who completed a 10-week email journey had high recall and varied emotional responses to heart age, including motivation, optimism, anxiety, and worry [14]. Recall was lower for equal heart age, and anxious or worried responses were higher for older heart age. Most of the respondents reported improved diet and exercise, with many reporting weight loss, reduced stress, and reduced smoking. People with older or equal heart age reported higher rates of diet and weight loss than those with younger heart age. People with older heart age were more likely to visit a physician or have a heart health check compared with those with younger or equal heart age. Credibility issues were identified in open responses. A UK study of consultations using 2 different risk communication tools found that 10% of consultation time (<2 minutes) was devoted to CVD risk [33]. Using JBS-3 increased the time spent discussing CVD risk, the proportion of patients asking questions about CVD risk, discussion of heart age, and medication, whereas using QRISK2 increased the time spent talking about risk factors. One-fifth of consultation time was spent on interventions, mostly lifestyle (Table 3).

### Mixed Methods Studies With Qualitative Analysis

Four studies used qualitative methods, including focus groups [34,35], interviews [35-37], think-aloud [36] and video prompt [37] methods. In general, the findings reflected trial outcomes in relation to recall, risk perception, emotional response, motivation, and credibility. Heart age was noted by participants across the studies as being more memorable and easier to understand than other risk formats. In a UK study where recorded consultation videos were used as a prompt for interviews, some practitioners did not fully understand risk percentages and preferred to use heart age or seemed more confident in discussing heart age as opposed to percentage risk [37]. This study also indicated that additional behavior change techniques may be added by the health provider depending on how they use the heart age tool. As reflected in the quantitative data, heart age also resulted in stronger emotional reactions from participants, for example, “it was the heart age that really shook me” [35]; however, it was noted that this could be either motivating or frightening [34]. Heart age prompted some participants to consider changing their lifestyle (eg, losing 9 kg [35]; “my weight is something I need to work on” [36]), which was also reflected in those who saw their risk percentage, and many with low or moderate risk scores discussed lifestyle changes. There was a suggestion that any intervention discussing risk with participants acts as a “reminder” or ”trigger,” “the kick that [they] needed” [35] to change behavior. Similarly, discussing risk with a practitioner resulted in the intention to change behavior [37]. Some participants were skeptical of the calculations for heart age and questioned their credibility (Table 3) [34,36,37].

### Behavior Change Techniques

**Figure 2:**
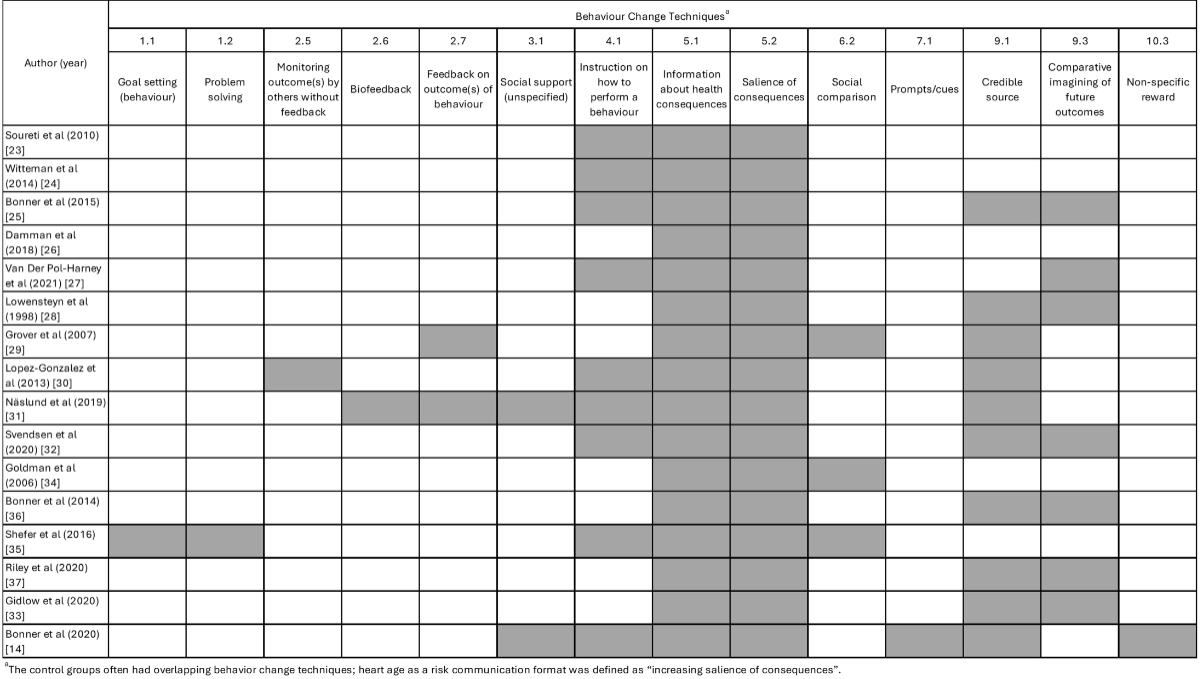
Behavior change techniques mentioned in methods for heart age interventions [14,23-37].

The interventions included in the heart age studies varied in terms of behavior change techniques. From the Michie et al taxonomy of 93 techniques [19], we identified 14 from methods section descriptions or published protocols. All studies included salience of consequences by definition (as we interpreted heart age as increasing the salience of the risk assessment) and information about health consequences (ie, CVD outcomes). Instructions on how to perform a behavior, credible source, and comparative imagining of future outcomes were common for both the intervention and control groups. It should be noted that the results sections of the qualitative studies indicated that additional behavior change techniques may be used by health providers using these tools in a consultation, and this may influence how effective they are in clinical practice (Figure 2 [14,23-37]; Multimedia Appendix 2).

### Risk of Bias Assessment

**Figure 3:**
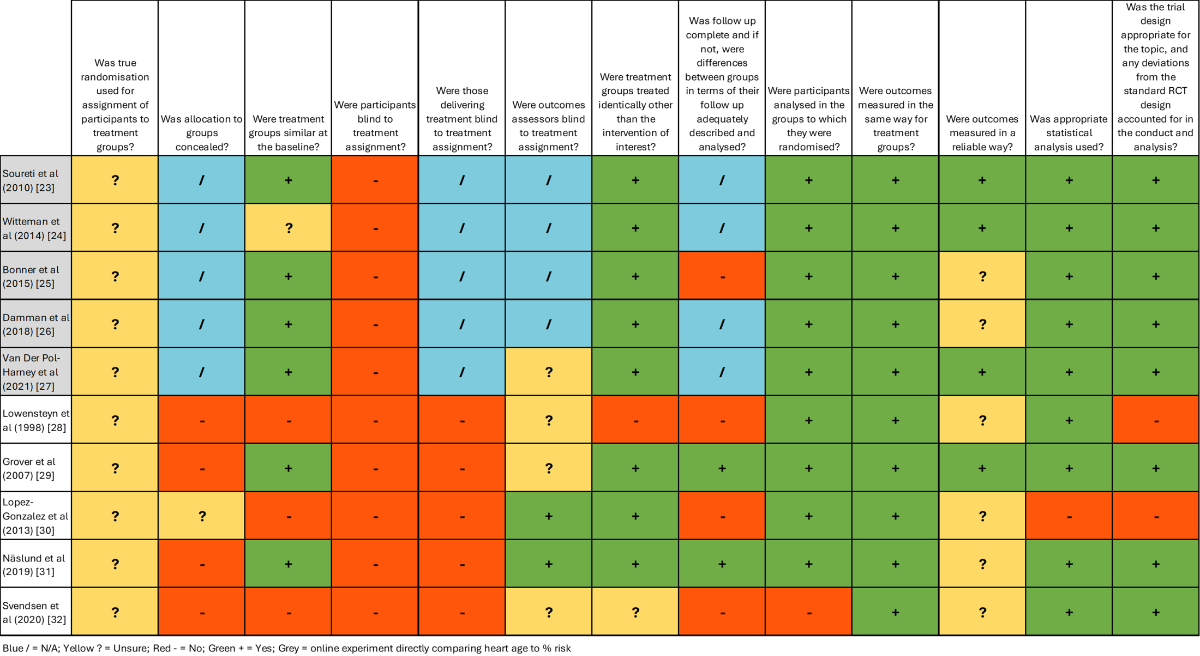
Risk of bias assessment for quantitative studies. RCT: randomized clinical trial [23-32].

Risk of bias was noted for all randomized studies, with some applied clinical trials being particularly concerning in terms of unclear or questionable methods for randomization and analyses, including contamination between groups and analysis not per original randomized group. All experimental trials used self-reported outcomes rather than objective methods (Figure 3, [23-32]).

## Discussion

### Principal Findings

When randomized trials are separated into direct comparisons between heart age and absolute risk versus complex interventions, there is limited evidence for the effectiveness of heart age over absolute risk expressed as a percentage risk over time in terms of lifestyle change. Heart age does appear to evoke a greater emotional response (both positive and negative), increases risk perception (although low-risk people may think they are high risk), and reduces credibility [23-27]. Both percentage risk and heart age can be effective as risk communication formats when combined with other various behavior change techniques in applied settings, and have the potential to reduce blood pressure, cholesterol levels, and weight, which in turn can reduce absolute risk [28-32]. Only one study [27] compared different labels for the heart age concept and found different psychological effects, indicating the importance of testing evaluative labels with the intended target audience. Qualitative and mixed methods studies generally reflected the outcomes measured in the randomized studies but tended to conclude that heart age was more motivating than percentage risk for lifestyle change, whereas randomized trial data did not support this assertion [14,33-37].

### Limitations

Owing to the heterogeneity of both the intervention components and the outcomes, we were unable to synthesize the results as a meta-analysis. Therefore, our findings are descriptive across a range of outcomes and measurement methods. All randomized studies had some risk of bias, particularly some of the complex intervention studies in applied settings where randomization and analysis processes did not follow best practices, including contamination between groups and analyses not reflecting the initial randomization to groups. Outcomes in experimental studies were based on self-report rather than objective measurements.

### Comparison With Previous Work

This is the first systematic review of the effects of heart age interventions. Previous reviews of CVD risk communication formats or biological age concepts have either been descriptive in relation to the models themselves [16,20] or have not differentiated between the behavior change techniques used in intervention versus control groups, leading to mixed results overall [17,38]. This study shows the importance of identifying active ingredients in behavioral interventions to identify meaningful comparisons for future reviews [19]. The design of applied clinical trials of heart age interventions did not differentiate between heart age as a risk communication format and supplementary behavior change techniques, with an insufficient description of the meaningful differences between the intervention and control groups. The finding in one study that the label for the heart age concept (heart age vs fitness age) affected outcomes echoes recent findings on different terms for elevated blood pressure [27,39].

### Future Research

The risk of bias could be improved in future heart age trials, but we note that blinding is not generally possible in a risk communication intervention. The mixed methods studies suggest additional outcomes should be included, such as overall consultation time, clinical communication content, and time spent on different aspects (eg, risk factors, risk communication formats, risk manipulation, lifestyle change, and medication [33,37]). Several studies highlight the importance of differentiating among older, younger, and equal heart age results in analyses and considering expectations in relation to this [14,25,27]. Authors of heart age studies need to take care to specify the components of their interventions, including the risk communication tool itself and the way that health care providers use and explain this in a consultation. Our findings suggest that the most appropriate outcomes to measure for heart age as a standalone risk communication format are emotional response, perceived credibility, and risk perception. Combining heart age with other behavior change techniques may be effective for behavior change if they are selected for a specific outcome in mind based on other evidence. Authors should avoid overstating the benefits of heart age as a standalone risk communication or behavior change tool by ensuring that all conclusions are supported by the data.

### Practical Implications

The appeal of heart age to consumers is suggested by its widespread use among millions of people worldwide [13-15]. However, if lifestyle change is the intended outcome, additional support is needed using evidence-based behavior change techniques, such as action plans and goal-setting. This is in line with the behavior change literature, where many different health models that show risk communication is necessary but not sufficient for behavior change. Heart age is a risk communication format that can capture attention and provoke an emotional response, but it is not enough as a standalone intervention for behavior change [40]. For example, in the Australian survey identified in this review, the initial heart age assessment was followed by a 10-week email journey where behavior change could be reinforced with prompts, activities and planning tools [14]. In a recent review on how to present probabilities in patient decision aids, biological age was not recommended because it may undermine understanding of absolute risk, which is required for making informed, shared decisions about medication [41]. General practitioners, nurses, and cardiologists engaged in CVD risk assessment need to consider what their communication aim is to determine whether heart age or absolute risk is most appropriate. If heart age is communicated with the aim of motivating lifestyle change, it needs to be supported by other behavior change techniques such as action plans.

### Conclusions

This review found little evidence that heart age motivates lifestyle behavior change more than percentage risk, but either format can improve clinical outcomes when combined with other behavior change strategies. The label for the *heart age* concept can affect outcomes and should be pretested with the intended audience. Future research should more carefully specify the components of the intervention, avoid overstating the benefits of heart age as a standalone risk communication format, consider effects on physician-patient consultations, and differentiate between older and younger heart age results.

## Appendix

Multimedia Appendix 1 Measures used in each study, and whether significant outcomes were found.

Multimedia Appendix 2 Behavior change techniques by intervention group.

## Abbreviations

CVD: cardiovascular disease
JBS: Joint British Societies
